# In Situ Computed Tomography—Analysis of a Single-Lap Shear Test with Clinch Points

**DOI:** 10.3390/ma14081859

**Published:** 2021-04-09

**Authors:** Daniel Köhler, Robert Kupfer, Juliane Troschitz, Maik Gude

**Affiliations:** Institute of Lightweight Engineering and Polymer Technology (ILK), Technische Universität Dresden, 01069 Dresden, Germany; robert.kupfer@tu-dresden.de (R.K.); juliane.troschitz@tu-dresden.de (J.T.); maik.gude@tu-dresden.de (M.G.)

**Keywords:** clinching, computed tomography, in situ CT, lap shear testing

## Abstract

As lightweight design gains more and more attention, time and cost-efficient joining methods such as clinching are becoming more popular. A clinch point’s quality is usually determined by ex situ destructive analyses such as microsectioning. However, these methods do not yield the detection of phenomena occurring during loading such as elastic deformations and cracks that close after unloading. Alternatively, in situ computed tomography (in situ CT) can be used to investigate the loading process of clinch points. In this paper, a method for in situ CT analysis of a single-lap shear test with clinched metal sheets is presented at the example of a clinched joint with two 2 mm thick aluminum sheets. Furthermore, the potential of this method to validate numerical simulations is shown. Since the sheets’ surfaces are locally in contact with each other, the interface between both aluminum sheets and therefore the exact contour of the joining partners is difficult to identify in CT analyses. To compensate for this, the application of copper varnish between the sheets is investigated. The best in situ CT results are achieved with both sheets treated. It showed that with this treatment, in situ CT is suitable to properly observe the three-dimensional deformation behavior and to identify the failure modes.

## 1. Introduction

Usually, clinch points are experimentally characterized by ex situ lap shear tests where the force–displacement behavior is monitored and damage phenomena such as pull-out (possibly with neck fracture), neck fracture, and neck fracture with plastic deformation (mixed mode failure) are investigated [1]. Additionally, in microsection analyses, the geometrical characteristics such as bottom thickness, neck thickness, and undercut are measured. While the undercut predominantly determines the pull-out and peeling strength, the neck thickness mainly influences the shear strength of a clinching point. Beyond that, the bottom thickness generally indicates the clinch point quality. The main disadvantage of ex situ analyses is that the observed damage state is inaccurate due to the resetting of elastic deformations and crack closure effects. On the contrary, non-destructive in situ measurement methods allow the online investigation of the material flow and damage chronology during testing. In ref. [2], the electrical resistance of aluminum–steel clinch points are investigated in situ, showing a characteristic relation of the electrical resistance to the punch force and the bottom thickness.

Another common non-destructive testing method is radiographic imaging, where X-rays are directed toward an object. The attenuated radiation is measured by the detector, creating a two-dimensional projection of the object (X-ray image) [3]. While radiography takes an X-ray image from a single angle, in computed tomography (CT), the object is scanned from several angles. Then, the set of projections is used to reconstruct a three-dimensional image of the object in high resolution [3].

CT is established for geometry and structural analyses, both of classical homogeneous materials (e.g., casted aluminum alloy [4]) and of heterogeneous material systems, such as concrete [5], composites [6], or biomaterials [6]. Primarily, it is used to identify the inner microscopic structure [7] or pores [4,5], but especially for textile composites, the analysis of delamination, fiber fracture, and ondulation [8] is important. In the field of joining technology, fiber-reinforced plastics (FRP) and aluminum (Al) joined by semi-tubular self-piercing rivets [9] and drilled holes [10] are investigated.

Recently, the CT method was extended for the investigation of joining and testing processes in situ. Füßel et al. used in situ CT for the damage analysis of single overlapping adhesively bonded riveted lap joints of FRP and Al in lap shear tests [11]. Kunz et al. presented a technique for measuring the internal displacement field in adhesives by tracking characteristic markers within adhesives during loading using in situ CT [12]. Furthermore, Pottmeyer et al. used in situ CT to investigate the damage evolution of carbon FRP due to the deformation of inserts under tensile loading. Here, the detachment of the boundary layer and the insert, fiber fracture, and delamination allocated to the respective forces were shown [13].

**Figure 1:**
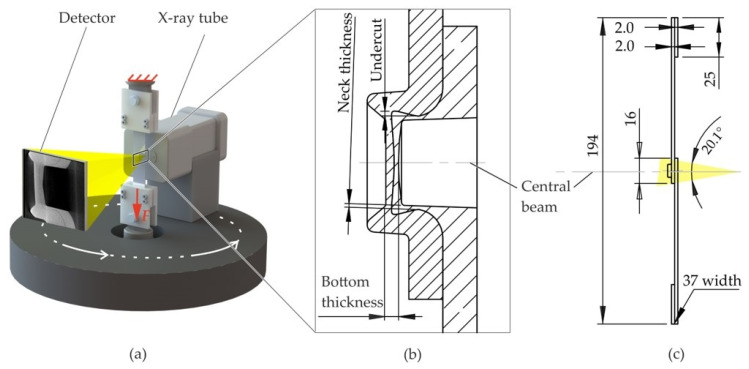
Schematic test set-up in the in situ computed tomography (CT) (**a**), the cross-section of the lap shear specimen in the initial position with the characteristic dimensions (**b**) and the overall specimen dimensions (in mm) (**c**).

The aim of this paper is to describe the potential of detecting deformation characteristics and damage phenomena of clinch points during single-lap tensile shear testing (lap shear test) using in situ CT. For that purpose, a tensile test set-up in combination with a CT system as shown in Figure 1 is used. Initial tests showed that the sheet–sheet interface in the clinch points neck cannot be identified properly in the CT analysis because the sheets are in close contact with each other. As this limits the potential of this method, a radiopaque layer is applied to the specimens to enhance the visibility of the interface. Earlier investigations showed that barium iodide solution and a tin foil are suitable as radiopaque interlayers [14]. However, the specimens applied with the solution exhibited an inconsistent coating, and the tin foil leads to artefacts and blurring as well as wrinkling of the foil. Therefore, in this paper, the application of a conductive copper varnish spray (copper spray) is investigated. The deformation characteristics and damage phenomena in the specimen measured with the new method are shown and clinch points with and without radiopaque layer are compared. A finite element (FE) model is set up to simulate the structural behavior of the specimen during lap shear testing. Comparing FE and in situ CT results, the potential for validation of numerical models is shown.

## 2. Materials and Methods

### 2.1. In Situ Lap Shear Test

The in situ lap shear test of the clinched specimen is based on ISO 12996 [1], where only the force–displacement curve is measured and the failure mode of the joint is determined after failure. In the current in situ lap shear test set-up, the specimen consists of two lubricated 2 mm thick sheets made of aluminum EN AW 6014-T4 (Advanz™ 6F-e170, Novelis Inc., Atlanta, GA, USA) that are clinched with the punch A50100 and the die BE8012 (both from TOX PRESSOTECHNIK GmbH & Co.KG, Weingarten, Germany) in the overlapping area. The characteristic dimensions in the cross-section of the joint are shown in Figure 1b. The specimen is solution heat-treated and artificially aged (T6) by 458.15 K over 20 min after clinching. In order to ensure a concentric load introduction, two shim plates are bonded onto the ends of the sheets. In contrast to the norm, the specimen width is 37 mm instead of 45 mm (cf. Figure 1c) to fit the clamps. The specimen is clamped on both sides in the in situ tensile testing machine based on a ZwickRoell Z250 (ZwickRoell GmbH & Co. KG, Ulm, Germany) and pulled along the main axis (lower traverse is moving).

The clinch point is CT-scanned at nine displacement steps whereby the crossbeam remains at constant displacement. The displacement is measured as the travel of the crosshead because direct measurements using e.g., extensometers with sensor arms are not compatible with an in situ CT measurement. The CT system FCTS 160-IS (FineTec FineFocus Technologies GmbH, Garbsen, Germany) from Finetec consists of the X-ray source FORE 160.01C TT (160E3 V, 1E-3 A, 80 W) and a flat panel detector (3200 × 2300 pixel, 405 mm × 290 mm active area). In this CT system, the X-ray-source-detector system rotates around the specimen in order to take the X-ray images from different angles.

The parameters for the CT, the lap shear test, and the specimen manufacturing are summarized in Table 1.

### 2.2. Enhancing the Detectability of the Sheet–Sheet Interface Using Radiopaque Materials

**Figure 2:**
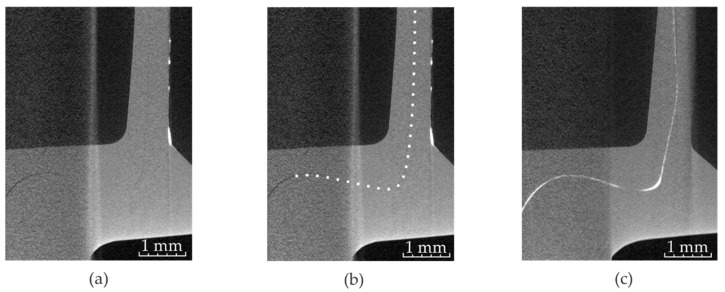
Detail of a computed tomography (CT) image of a clinch point with poorly visible sheet–sheet interface (**a**) and the same CT image with the assumed interface marked with a white dotted line (**b**). The same detail with copper varnish (applied on both sheets) in the sheet–sheet interface (**c**).

Preliminary CT scans of clinch points showed that the interface between the two sheets is hard to identify especially at the clinch point’s neck. Since the sheet’s surfaces are pressed excessively together, the X-rays attenuation does not deviate significantly enough from the attenuation by a homogenous aluminum material. Consequently, the undercut and the neck thickness are hardly measurable (cf. Figure 2a,b). Since solved barium iodide and the thin tin sheet did not lead to sufficiently good results, copper spray is investigated. Initial tests on two 20 mm × 20 mm × 2 mm clinched aluminum sheets revealed a very consistent and well visible layer in the sheet–sheet interface (cf. Figure 2c).

For the lap shear test, the sheets are sprayed with Tifoo conductive copper varnish (MARAWE GmbH & Co. KG, Regensburg, Germany) spray with a distance of approximately 200 mm at the overlapping area and then left for drying at room temperature. In order to investigate the effect of the layer on the visibility of the sheet–sheet interface and on the clinching and testing process, lap shear tests are conducted with specimens with copper spray applied to neither of the sheets, to one sheet, and to both sheets (cf. Table 2). The influence of the copper spray on the clinch point is investigated in in and ex situ CT investigations. In order to investigate the in situ lap shear test, three specimens with varying copper spray treatment are used.

In ref. [15], clinch points are manufactured with the same clinching parameters as described in Table 1 and the same sheet material but without any radiopaque interlayer. The characteristic dimensions measured in a micro-section are given. In this paper, the results are used for validation of the clinching simulation and for the investigation of the influence of the radiopaque material on the clinch point characteristic dimensions.

### 2.3. Numerical Model

**Figure 3:**
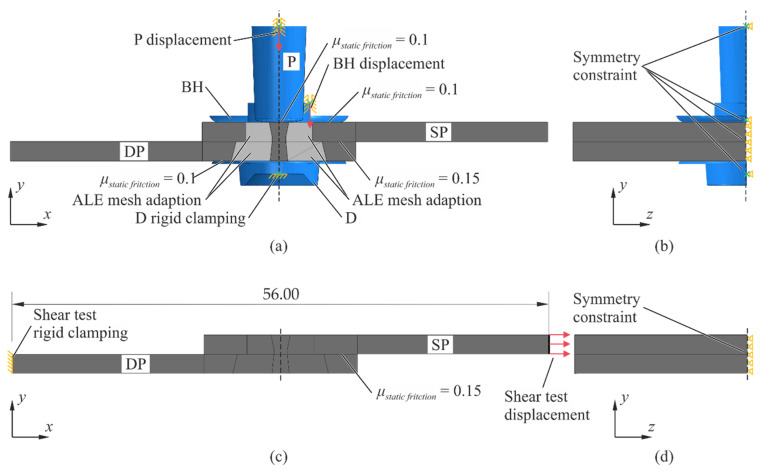
Set-up of the 3D numerical model for the clinching process (**a**,**b**) and for the lap shear test (**c**,**d**). The sheets (gray), the areas of Arbitrary Lagrangian–Eulerian (ALE) mesh adaption (light gray) and the tools (blue) are shown.

In order to compare the in situ CT tests with numerical simulations, both the clinching process and the lap shear testing are simulated (Abaqus 2017, explicit). While for clinching simulation (round point), a 2D axisymmetric model is often suitable [16], the lap shear process has only a single symmetry plane requiring a 3D model. However, to avoid inaccuracies due to the transfer of the material states between these models, both processes are set up as a 3D model with the symmetry *y*-*z* plane applied (cf. Figure 3b,d). In order to decrease the computational effort, the punch (P), the die (D), and the blank holder tip (BH) are modeled as rigid solids, and the punch-faced sheet (SP) and die-faced sheet (SD) are modeled as deformable solids. In order to assess this assumption, a reference axisymmetric 2D (reference model) model was set up where the tools are designed with the same dimensions but as deformable solids. The evaluation found that using rigid solids in the 3D model, which is a common adaption [17,18], leads to negligible deviations from the reference model.

**Figure 4:**
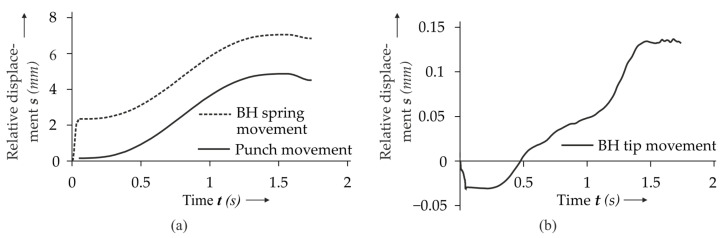
Blank holder spring compression and punch movement (**a**) and the resulting blank holder tip movement (**b**).

The numerical model of the clinching process with the boundary conditions and loads is shown in Figure 3a,b. The die is fixed and the punch is displaced in a smooth curve (cf. Figure 4a). It penetrates the sheet stack by 4.71 mm and subsequently retracts by 0.2 mm. In the experiment, the BH is mounted to a spring. Designing the spring in the 3D model can lead to inaccuracies due to the application of artificial mass scaling; i.e., the BH can be affected by the increased inertia forces. In order to avoid this, an auxiliary axisymmetric 2D model (auxiliary model) is used to specify the displacement behavior of the BH. The parts in this model have the same dimensions as in the reference model, but the tools are designed as rigid solids. Additionally, a spring element and negligible artificial mass scaling is used. The spring, having a stiffness of 485 N/mm^2^, is initially precompressed to 1060 N. Then, it compresses according to the displacement curve of the punch (cf. Figure 4a). The resulting displacement curve of the BH (cf. Figure 4b) is used as displacement constraint of the BH in the 3D model.

The numerical model of the lap shear test with its boundary conditions and loads is shown in Figure 3c,d. The specimen has a free length of $l_{Experiment}=144\;\mathrm{mm}$. In order to decrease computational effort, the specimen is modeled up to the length where the von Mises stresses caused by the clinch process remain above 10% of the maximum stresses (tested with the auxiliary model). Thus, a length of $l_{Simulation}=56\;\mathrm{mm}$ of the specimen is modeled (cf. Figure 3c). Consequently, the relation between experimental

Displacements $u_{Experiment}$ and numerical displacements $u_{Simulation}$ is

(1) $$u_{Experiment}=u_{Simulation}+\frac{F_{Simulation}}{t\cdot b\cdot E}\cdot \left(l_{Experiment}-l_{Simulation}\right).$$

Here, $F_{Simulation}$ is the measured tensile force in the simulation, t and b are the sheet thickness and width, and E is the Young’s modulus. After retraction of the punch, interactions between the tools and the sheets are disabled. Simulating the lap shear test, the die-faced sheet is fixed, and the punch-faced sheet is displaced by 0.7 mm over 22 s in a smooth curve.

The sheets aluminum material is EN AW-6014 [15], and the tool steel is the X 155 CrVMo 12 1 [19] (used in the reference model), which is a common tool steel for punches and dies [19]. Both materials are modeled as elasto-plastic materials. In order to avoid excessive element distortion during the virtual clinching process, Arbitrary Lagrangian–Eulerian (ALE) adaptive mesh is assigned to the highly deformed domain of the sheet meshes (cf. Figure 3a, light gray area). A remeshing frequency of 25, and three remeshing sweeps are chosen. The 3D model consists of 195,000 elements including 170,000 linear hexahedral elements with reduced integration (C3D8R) in the two sheets.

The interactions between the solids are defined as surface-to-surface contacts with a kinematic contact method. Only the contact between the blank holder tip and upper sheet is constrained with the penalty contact method. Coulomb’s friction model is used, but the kinetic friction coefficient is considered negligibly low because the tool surfaces are polished. The maximum shear stress from which slipping occurs is estimated with $\tau_{max}=\;\sigma_{yield}/\sqrt{3}$ [20]. Using the lower yield strength of the aluminum sheet $\sigma_{yield}=100\;\mathrm{MPa}$, a maximum shear stress of $\tau_{\max}=\;58\;\mathrm{MPa}$ is calculated.

As described in ref. [21], the literature data for aluminum pairings deviate strongly. Hence, the static friction coefficients are determined iteratively to fit the model to the characteristic dimensions given in ref. [15]. Thus, a friction coefficient of 0.1 between the tools and the sheet and a friction coefficient of 0.15 between the sheets is applied.

## 3. Results

### 3.1. Numerical Model Validation

For validation of the clinching model, the results of the simulation and of [15] are compared in Table 3. It shows that the simulation and the experiment agree well concerning the maximum clinching force and the characteristic dimensions. During simulation, the kinetic to internal energy ratio remains below 0.1%, justifying the artificial mass scaling.

### 3.2. Influence of the Radiopaque Materials

**Figure 5:**
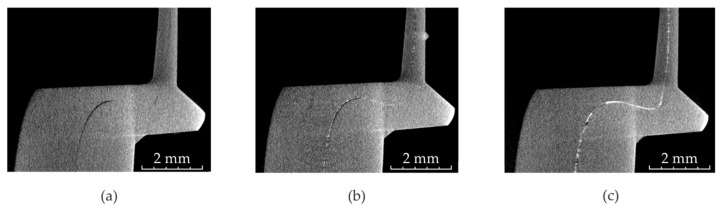
Half CT image of the specimens with copper spray applied to neither of the sheets (**a**), to one sheet (**b**) and to both sheets (**c**).

The influence of the copper spray on the detectability of the interface can be seen in Figure 5. Only the specimen with both sheets treated show an almost consistent layer of copper spray (cf. Figure 5c). The characteristic dimensions of the non-treated and treated specimens measured in CT are summarized in Table 4. In the CT measurement, two specimens were investigated each at five locations. Here, no noticeable influence of the interlayer on the characteristic dimensions can be detected. However, the measurement of the undercut and the neck thickness is still accompanied with higher uncertainties for the specimen treated only on one sheet due to the poorly visible interface (cf. Figure 5b).

**Figure 6:**
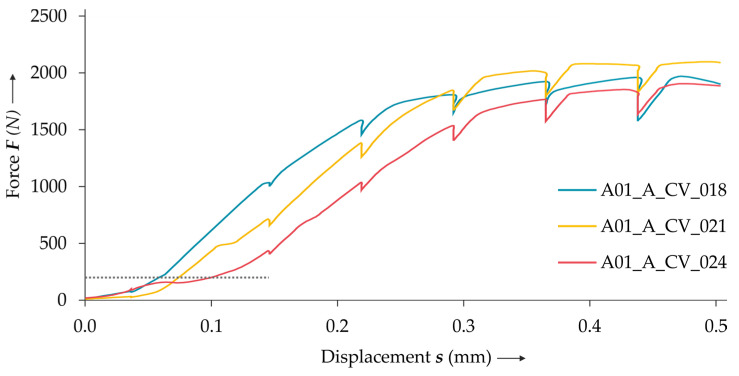
The results of the lap shear tests. The baseline value 200 N is marked with a dotted line.

The results of the in situ CT lap shear tests show that there is displacement accompanied with low forces at the beginning of the test (cf. Figure 6). This is due to alignment and setting effects in the clamps and the adapters of the tensile testing machine. As these effects have largely faded at 200 N (cf. Figure 6), the lap shear results of the numerical and the in situ investigation are shifted toward this baseline value. Additionally, the displacement due to the machine rigidity of the tensile testing machine is removed via preliminary calibration tests. In the diagram, a drop of the force at the nine displacement steps is clearly visible, which is caused by relaxation effects in the test set-up.

**Figure 7:**
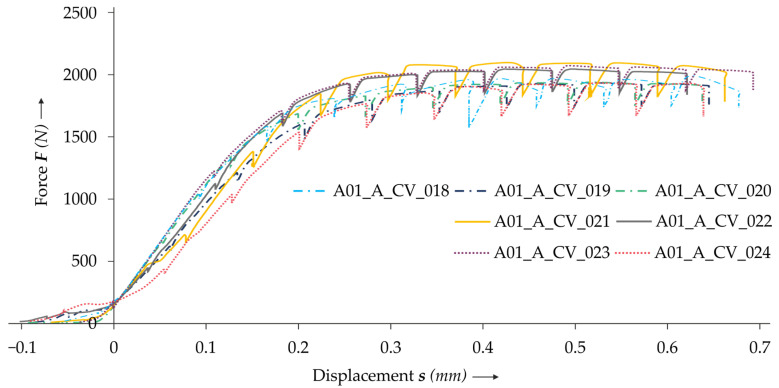
The results of the lap shear tests using specimens with one sheet treated (dash-dot line), with both sheets treated (continuous line) and without treatment (dotted line).

Comparing the results of the lap shear tests of the non-treated and treated specimens, no clear influence of the radiopaque material can be seen (cf. Figure 7).

**Figure 8:**
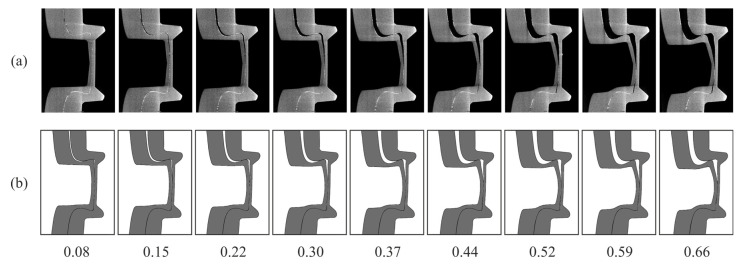
Comparison of (**a**) the cross-section of the in situ CT lap shear test (both sheets treated) with (**b**) the simulation at the investigated displacements (in mm).

As only the specimen with both sheets treated provides a sufficient sheet–sheet interface detectability (cf. Figure 5), specimen A01_A_CV_018 is used for the comparison with the numerical model. In Figure 8, the reconstructed cross-sections (top) of the in situ CT lap shear test (both sheets treated) are compared with the simulation results (bottom) at the investigated displacements. At the beginning of the investigation, the punch sided sheet lifts from the other sheet, resulting in an increasing gap between both sheets. During this movement, both sheets interact in the neck area, and the punch-sided sheet slides in the z-direction, leading to unbuttoning. It also can be observed that the straight contour of the indention of the punch-faced sheet increasingly deforms to an S-shaped curve. As a result of the radiopaque layer, the sheet–sheet interface remains visible throughout the whole investigated process. Thus, the deformation in the clinch point, especially the thinning of the neck, can clearly be identified. Furthermore, it can be stated that the in situ CT reconstructions show good agreement with the numerical results.

**Figure 9:**
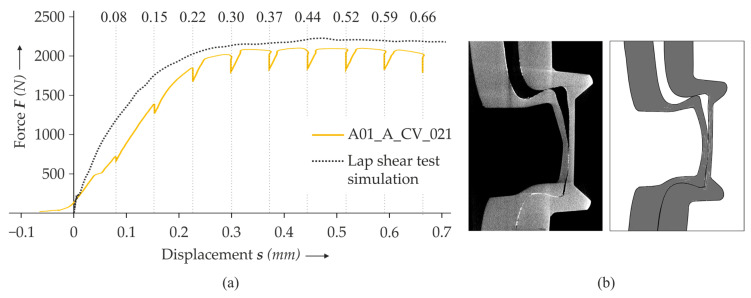
Comparison of the force–displacement curve of the in situ CT lap shear test (using a specimen with both sheets treated) with the simulation at the investigated displacements (in mm) (**a**). The comparison of the cross-section of the in situ CT lap shear test (both sheets treated) with the simulation at 0.66 mm displacement in detail (**b**).

The corresponding force–displacement curves are displayed in Figure 9a. They show a good qualitative agreement with the experiment. Comparing the cross-section of the in situ CT result and the simulation in detail in Figure 9b shows that the geometrical characteristics during unbottening also align well in the simulation and experiment. However, the stiffness of the specimen in the simulation seems to be slightly higher than in the simulation, and the predictability below 0.2 mm needs to be improved.

## 4. Discussions

In the center of the investigations stands the in situ CT lap shear test of the clinched EN AW-6014 specimen. In addition to principle feasibility, a new method was tested to make the interface between both sheets in the joining zone more visible. While other work concentrates on the dark field imaging method [22] to enhance the contrast between different volumes, in the current work, a thin interlayer is introduced prior to joining. Although this concept was already tested in ref. [14] by using barium iodide or tin interlayers, it could be improved significantly. It shows that the major drawbacks of tin foils (folding and strong artefacts) as well as barium iodide solution treatment (low visibility) can be compensated by a double application of copper spray.

However, the introduction of an additional interlayer changes the tribological system in the joining zone. This harbors the danger that the joining process is significantly influenced by the radiopaque interlayer and the resulting characteristic geometry differs from untreated joints. Hence, the characteristic values of treated specimen are compared with the results of other research groups. In Table 3 (line 1), the microsection measurements from ref. [15] are given. They used the same clinching setup and material configuration such as the present study. The measured undercut, bottom, and neck thickness values of treated specimens in Table 4 (lines 2 and 3) show a very good agreement with the non-treated microsection results. This can be seen as a first quantitative confirmation of the approach. Further studies could be performed to clear the slight differences for the bottom thickness between CT and microsectioning.

The numerical model was set up following technics used in previous publications. The tools were designed as rigid solids agreeing with ref. [17] and ref. [18]. Additionally, an elasto-plastic material, mesh adaption, and Coulomb friction were modeled, which is a common method according to ref. [16]. Finally, the results of the numerical clinching investigation agree well with [15]. Concerning the lap shear simulation, the holding of the constant displacement of the specimen and consequently the drop of force is not considered due to the complex and unknown setting effects in the test set-up. Neglecting the kinetic friction and estimating the maximum shear stress is a first approach to model the clinching process and the lap shear test. However, describing the model with empirical data can enhance the quality of the numerical results.

To the best of the authors’ knowledge, there has been no publication about investigations of lap shear tests of clinch points in in situ CT. The results show the potential of an in situ investigation of joining processes using CT. However, in order to increase the relevance of the method, a better CT image quality is necessary. This could be achieved by increasing the magnification and reducing the noise level. The former can be realized by decreasing the distance between the X-ray source and specimen, which would require a specimen extension to a length of 210 mm. The latter can be achieved by reducing the specimen width so that less acceleration voltage is needed to penetrate the specimen. Furthermore, noise can be reduced by frame averaging.

The observed relaxation phenomenon, resulting in the drop of force before each CT-scan, is an inherent limitation of this method. Nevertheless, given that the force drop is limited, it can be expected that most of the elastic effects can be detected with this method. A quantitative comparison with classic ex situ measurements should be investigated further in the future.

## 5. Conclusions

In this paper, a lap shear test of clinched metal sheets is conducted using in situ CT. In order to support the detectability of the sheet–sheet interface, the specimens are treated with a copper spray in varying intensities. Furthermore, an FE model is set up to demonstrate the potential for the validation of numerical simulations by in situ measurements in the field of joining. Moreover, the deformation characteristics in the specimen measured with the new method are analyzed.

The results show that a lap shear test of a clinched specimen can successfully be conducted in in situ CT. By application of copper spray as a radiopaque layer, the visibility of the sheet–sheet interface is enhanced significantly, making the deformation process well traceable. The results of the lap shear test and of the numerical investigation agree well qualitatively. Improving the quantitative agreement should be the focus of future work. Here, the experimentally observed setting effects could be reduced by applying higher initial force. Furthermore, the displacement could be measured directly on the specimen using markers that are visible in the CT image.

Above that, the influence of the copper spray on the material flow in the clinching process and the testing needs to be investigated more accurately. The shearing between the sheets led to an insufficient copper varnish layer in the clinch point bottom. Therefore, a more intense treatment in this area should be investigated. Furthermore, systematic studies on the influence of the interface layer should be conducted with a higher amount of specimens.

## Figures and Tables

**Table 1:** Test and specimen manufacturing parameters.

Test Division	Parameter	Unit	Value
Specimen	Punch penetration	mm	4.7
	Joining speed	mm/s	2
	Heat treatment temperature (T6)	K	458.15
	Heat treatment duration (T6)	min	20
CT system	Acceleration voltage	V	1.50 × 10^5^
	Tube current	A	7.0 × 10^−5^
	X-ray projections	-	1440 (4 per 1°)
	Exposure time	s	6.25
	Resolution	mm	7.58 × 10^−3^
	Magnification	-	16.8
In situ testing machine	In situ CT elongation steps	mm	0.08; 0.15; 0.22; 0.30; 0.37; 0.44; 0.52; 0.59; 0.66
	Test speed	mm/min	2

**Table 2:** Specimen overview.

Specimen	Copper Spray Treatment	Investigation Method
A01_A_CV_018	One sheet treated	In situ CT lap shear test
A01_A_CV_019	One sheet treated	Ex situ CT and lap shear test
A01_A_CV_020	One sheet treated	Ex situ CT and lap shear test
A01_A_CV_021	Both sheets treated	In situ CT lap shear test
A01_A_CV_022	Both sheets treated	Ex situ CT and lap shear test
A01_A_CV_023	No treatment	Ex situ CT and lap shear test
A01_A_CV_024	No treatment	In situ CT lap shear test

**Table 3:** Results of the numerical analyses compared to the force and microsection measurement in ref. [12] (five specimens, no measurement uncertainty given).

Measurement Method	Max. Clinching Force (N)	Bottom Thickness (mm)	Undercut (mm)	Neck Thickness (mm)
Microsection measurement [12]	3.25 × 10^4^–3.35 × 10^4^	0.67–0.72	0.25–0.31	0.45–0.52
Reference model	3.20 × 10^4^	0.68	0.31	0.43
Auxiliary model	3.09 × 10^4^	0.68	0.30	0.44
3D model	3.12 × 10^4^	0.68	0.28	0.45

**Table 4:** The results of the CT measurement of differently treated specimens.

Copper Spray Treatment	Bottom Thickness (mm)	Undercut (mm)	Neck Thickness (mm)
Not treated	0.65	unidentifiable	unidentifiable
One sheet treated	0.64	0.23–0.33	0.45–0.50
Both sheet treated	0.64	0.23–0.27	0.45–0.49

## Data Availability

The data presented in this study are available on request from the corresponding author.

## References

[1] International Organization for Standardization (2013). ISO 12996:2013(en), Mechanical Joining—Destructive Testing of Joints—Specimen Dimensions and Test Procedure for Tensile Shear Testing of Single Joints.

[2] Jiang T., Liu Z.-X., Wang P.-C. (2015). Quality inspection of clinched joints of steel and aluminum. Int. J. Adv. Manuf. Technol..

[3] Carmignato S., Dewulf W., Leach R. (2018). Industrial X-ray Computed Tomography.

[4] Nicoletto G., Konečná R., Fintova S. (2012). Characterization of microshrinkage casting defects of Al–Si alloys by X-ray computed tomography and metallography. Int. J. Fatigue.

[5] Du Plessis A., Olawuyi B.J., Boshoff W.P., Le Roux S.G. (2016). Simple and fast porosity analysis of concrete using X-ray computed tomography. Mater. Struct..

[6] Böhm R., Stiller J., Behnisch T., Zscheyge M., Protz R., Radloff S., Gude M., Hufenbach W. (2015). A quantitative comparison of the capabilities of in situ computed tomography and conventional computed tomography for damage analysis of composites. Compos. Sci. Technol..

[7] Jones A.C., Arns C.H., Sheppard A.P., Hutmacher D.W., Milthorpe B.K., Knackstedt M.A. (2007). Assessment of bone ingrowth into porous biomaterials using MICRO-CT. Biomaterials.

[8] Holub W., Haßler U. Detection and Evaluation of Ondulations in Glass-Fiber Reinforced Materials. Proceedings of the 4th Conference on Industrial Computed Tomography (iCT).

[9] Drossel W.G., Mauermann R., Grützner R., Mattheß D. (2013). Numerical and Experimental Analysis of Self Piercing Riveting Process with Carbon Fiber-Reinforced Plastic and Aluminium Sheets. KEM.

[10] Pejryd L., Beno T., Carmignato S. (2014). Computed Tomography as a Tool for Examining Surface Integrity in Drilled Holes in CFRP Composites. Procedia CIRP.

[11] Füßel R., Gude M., Mertel A. In-situ X-ray computed tomography analysis of adhesively bonded riveted lap joints. Proceedings of the 17th European Conference on Composite Materials.

[12] Kunz H., Stammen E., Dilger K. (2017). Local displacement measurements within adhesives using particle tracking and In Situ computed tomography. J. Adhes..

[13] Pottmeyer F., Bittner J., Pinter P., Weidenmann K.A. (2017). In-Situ CT Damage Analysis of Metal Inserts Embedded in Carbon Fiber-Reinforced Plastics. Exp. Mech..

[14] Köhler D., Kupfer R., Troschitz J., Gude M. Clinching in In-situ CT–Experimental Study on Suitable Tool Materials. Proceedings of the 24th International Conference on Material Forming (ESAFORM 2021).

[15] Bielak C.R., Böhnke M., Beck R., Bobbert M., Meschut G. (2021). Numerical analysis of the robustness of clinching process considering the pre-forming of the parts. J. Adv. Join. Process..

[16] Eshtayeh M.M., Hrairi M. (2016). Recent and future development of the application of finite element analysis in clinching process. Int. J. Adv. Manuf. Technol..

[17] Kaðèák L., Spiðák E., Kubík R., Mucha J. (2017). Finite Element Calculation of Clinching with Rigid Die of Three Steel Sheets. Strength Mater..

[18] Kaščák L., Mucha J., Spišák E., Kubík R. (2017). Wear Study of Mechanical Clinching Dies During Joining of Advanced High-Strength Steel Sheets. Strength Mater..

[19] Knörr M. (1996). Auslegung von Massivumformwerkzeugen Gegen Versagen Durch Ermüdung.

[20] Dassault Systémes Simulia Corp Abaqus Users Manual 2017: Version 2017, Providence, RI. https://abaqus-docs.mit.edu/2017/English/SIMACAEITNRefMap/simaitn-c-friction.htm.

[21] Köhler D., Kupfer R., Gude M. (2020). Clinching in in-situ CT—A numerical study on suitable tool materials. J. Adv. Join. Process..

[22] Cong W., Pfeiffer F., Bech M., Bunk O., David C., Wang G. (2010). Dark-field Tomography: Modeling and Reconstruction. https://arxiv.org/pdf/1003.2155.

